# Research progress on sarcopenia in the musculoskeletal system

**DOI:** 10.1038/s41413-025-00455-8

**Published:** 2025-09-23

**Authors:** Xinning Mao, Ke Lv, Weihui Qi, Wenqiang Cheng, Tenghui Li, Yueli Sun, Hongting Jin, Hao Pan, Dong Wang

**Affiliations:** 1 Department of Orthopaedics, Hangzhou TCM Hospital Affiliated to Zhejiang Chinese Medical University (Hangzhou Hospital of Traditional Chinese Medicine), Hangzhou, Zhejiang PR China; 2 Department of Orthopaedics, Hangzhou Dingqiao Hospital, Hangzhou, Zhejiang PR China; 3 Institute of Orthopaedics and Traumatology, Hangzhou Traditional Chinese Medicine Hospital Affiliated to Zhejiang Chinese Medical University, Hangzhou Traditional Chinese Medicine Hospital Affiliated to Zhejiang Chinese Medical University, Hangzhou, Zhejiang PR China; 4 Longhua Hospital, Shanghai University of TCM, Shanghai, China; 5 Institute of Orthopaedics and Traumatology, The First Affiliated Hospital of Zhejiang Chinese Medical University, Hangzhou, Zhejiang PR China

**Keywords:** Bone quality and biomechanics, Metabolic disorders

## Abstract

Sarcopenia, a progressive and systemic skeletal muscle disorder marked by the accelerated deterioration of both muscle function and mass, is highly prevalent among the elderly population, significantly contributing to an elevated risk of adverse outcomes, including falls, fractures, and muscle weakness. Clinical investigations have identified a strong correlation between sarcopenia and several prevalent degenerative skeletal muscle disorders. This correlation is attributed to imbalances in joint mechanics resulting from localized muscle atrophy and the influence of musculoskeletal secretory factors. In this review, we discuss the broader implications of sarcopenia and critically evaluate the currently established assessment methods. Furthermore, the clinical significance of prevalent musculoskeletal disorders (including osteoporosis, osteoarthritis, and spinal pathologies) in relation to sarcopenia, alongside the underlying mechanisms influencing this relationship, is summarized. Additionally, the effects of sarcopenia on the therapeutic efficacy of medications and surgical interventions for musculoskeletal conditions are reviewed. Sarcopenia is intricately linked to the onset, progression, and prognosis of musculoskeletal disorders. Future research should prioritize elucidating the potential mechanisms that connect muscle loss with skeletal muscle diseases, and investigating whether mitigating sarcopenia symptoms could decelerate the progression of these disorders, thereby paving new pathways for therapeutic interventions.

## Introduction

Sarcopenia is a progressive and generalized skeletal muscle disorder, characterized by a rapid deterioration in both muscle function and mass, which contributes to a heightened risk of adverse outcomes, including impaired physical function, increased falls, fractures, muscle weakness, and even mortality.^1^ Firxst introduced in the 1980s, sarcopenia was described as “an age-related decline in lean body”, with significant implications for mobility, nutritional status, and personal autonomy.^2^ The World European Working Group in Older People 2 (EWGSOP2) defines sarcopenia as a “progressive and generalized loss of skeletal muscle mass and strength,” highlighting the critical role of muscle strength deterioration.^1^ They further recommend that muscle strength be regarded as the most reliable measure of muscle function. Epidemiological data indicate that sarcopenia affects ~10%–16% of the global elderly population, with a notably higher prevalence (18%–66%) among those with underlying medical conditions such as diabetes, degenerative lumbar spine disease, cancer, or critical illness.^3^ Estimates of sarcopenia prevalence among community-dwelling older adults globally vary between 10% and 27%, with prevalence ranging from 8% to 36% in individuals under 60, and 10% to 27% in those over 60, according to sarcopenia working groups in Europe, Asia, and the United States, based on regional statistics.^4^ The observed heterogeneity in sarcopenia prevalence rates likely reflects inconsistencies in operational definitions and diagnostic approaches among different countries or regions.

Sarcopenia can be classified into primary (age-related) and secondary forms, depending on whether aging is the sole contributing factor or if additional pathological or lifestyle-related factors are involved.^5^ Secondary sarcopenia arises from comorbidities (e.g., cachexia, malnutrition, obesity) that adversely affect muscle mass and function, as well as from disuse atrophy due to chronic pain, musculoskeletal disorders, or prolonged sedentary behavior.^6^ The pathophysiology of sarcopenia involves multiple interrelated mechanisms, including chronic low-grade inflammation, vascular dysfunction, mitochondrial impairment, reduced satellite cell (myogenic progenitor) activity, disrupted muscle protein homeostasis, anabolic resistance, and neuromuscular degeneration.^5,7–9^ Age-related skeletal muscle remodeling is characterized by a shift from fast-twitch (type II) to slow-twitch (type I) fiber composition, accompanied by progressive intramuscular and intermuscular fat infiltration (myosteatosis) and a reduction in type II fiber-associated satellite cell populations.^10^ In addition, Insulin resistance secondary to metabolic dysregulation (e.g., diabetes and dyslipidemia) compromises skeletal muscle glucose metabolism while enhancing ectopic lipid deposition through increased cellular uptake of triglycerides and free fatty acids.^11,12^ Notably, several pathogenic mechanisms contributing to sarcopenia share common features with degenerative musculoskeletal disorders, including chronic low-grade inflammation, mitochondrial dysfunction, ectopic fat deposition, and metabolic dysregulation.

**Fig. 1:**
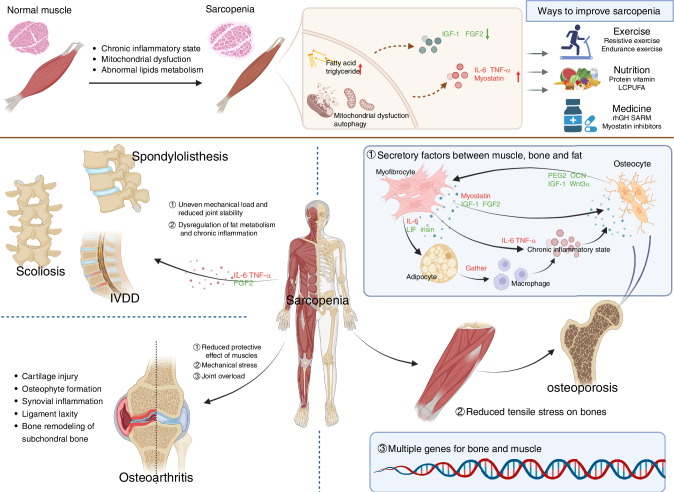
The association of sarcopenia with musculoskeletal disorders (Created in BioRender. Mao, X. (2024) https://BioRender.com/l97t655)

The human musculoskeletal system, comprising bones, cartilage, ligaments, tendons, joints, muscles, connective tissues, and other associated structures, plays a critical role in ensuring the normal functioning of life processes, movement, and various bodily activities. With advancing age, localized and systemic degenerative lesions of the musculoskeletal system, often resulting from mechanical injury and metabolic dysfunctions, significantly impact the quality of daily life and contribute to the increased risk of adverse outcomes in the elderly population. Clinical studies have revealed that a substantial proportion of patients with musculoskeletal disorders experience varying degrees of muscle loss, which is closely linked to musculoskeletal pain, low back pain, and other associated symptoms.^13–18^ As research on sarcopenia advances, growing evidence highlights shared pathological mechanisms between sarcopenia and musculoskeletal disorders, driving increased investigation into their common etiological factors and bidirectional interactions. This review summarizes the correlations between sarcopenia and prevalent musculoskeletal disorders (Fig. 1), focusing on clinical prevalence, underlying pathogenesis, treatment strategies, and prognosis. Investigating the influence of sarcopenia on musculoskeletal diseases may unlock novel avenues for mitigating the progression of these disorders.

## Diagnosis, evaluation, and management of sarcopenia

The variability in outcomes from sarcopenia assessments and diagnostics can be attributed to the diverse definitions of sarcopenia and diagnostic tools employed in studies across different regions and research settings. The EWGSOP2 definition of sarcopenia, which remains the most widely recognized standard, utilizes muscle strength and muscle mass assessments as key diagnostic indicators.^1^ The EWGSOP 2018 operational definition of sarcopenia is as follows: (1) Reduced muscle strength; (2) Decreased muscle mass or impaired muscle quality; (3) Diminished physical performance. If criterion 1 is met, sarcopenia should be suspected. Meeting criterion 2 confirms a diagnosis of sarcopenia. Severe sarcopenia is diagnosed when all three criteria (1, 2, and 3) are fulfilled. A comprehensive review of 283 studies addressing sarcopenia definitions up until 2015 revealed that 264 studies (93.3%) relied on muscle mass measurements to define sarcopenia, while 198 studies (70%) employed low muscle mass (LMM) as the sole diagnostic criterion.^19^ Among these studies, 43.6% employed dual energy X-ray absorptiometry (DEXA) as the primary technique for muscle mass measurement.^19^ Other commonly utilized methods for muscle measurement included bioelectrical impedance analysis (19.3%) and the L3 psoas major cross-sectional index (calculated as the total bilateral area of the psoas major muscles divided by the square of body height, as captured on a computed tomography (CT) axial slice at the L3 vertebral level) (13.6%).^19^

**Fig. 2:**
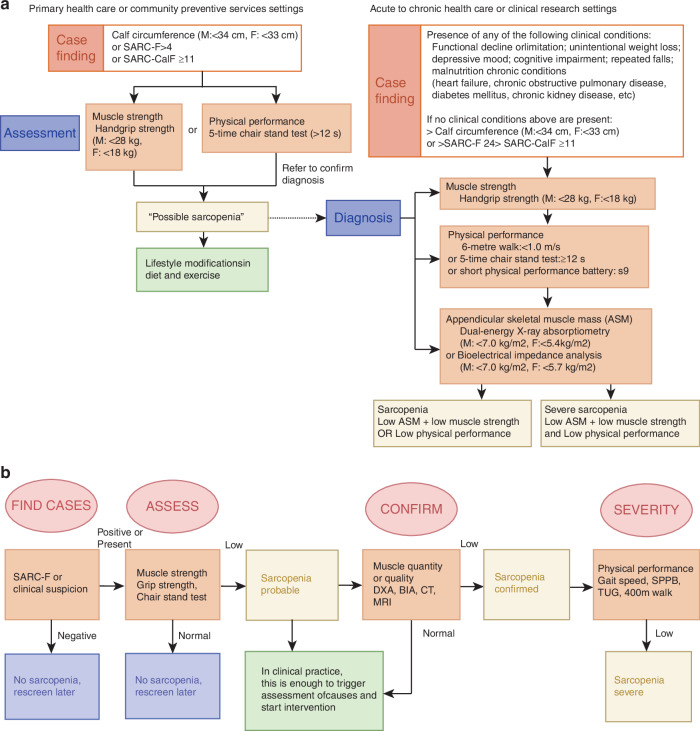
Diagnostic Algorithms for Sarcopenia from AWGS 2019 and EWGSOP2 Consensus Guidelines. **a** Diagnostic algorithm for sarcopenia recommended by the Asian Working Group for Sarcopenia (AWGS 2019 consensus guidelines). **b** Diagnostic algorithm for sarcopenia from the revised European Working Group on Sarcopenia in Older People consensus (EWGSOP2)

We evaluated the diagnostic algorithms for sarcopenia established by four major consensus groups: the European Working Group on Sarcopenia in Older People 2 (EWGSOP2), the International Working Group on Sarcopenia (IWGS), the Foundation for the National Institutes of Health (FNIH), and the Asian Working Group for Sarcopenia (AWGS).^1,20–22^ The International Working Group on Sarcopenia provided an initial framework for clinical assessment. In contrast, the Foundation for the National Institutes of Health (FNIH) guidelines emphasize the importance of differential diagnosis, requiring exclusion of weakness attributable to other medical conditions. More recently, the updated European Working Group on Sarcopenia in Older People 2 (EWGSOP2) and Asian Working Group for Sarcopenia (AWGS) consensus statements have introduced more comprehensive and detailed diagnostic algorithms for sarcopenia evaluation. The EWGSOP2 proposes a stepwise diagnostic approach incorporating muscle strength, mass, and physical performance measures with severity grading. In contrast, the AWGS recommends a two-tiered assessment framework: (1) an initial screening tool for community or clinical settings, and (2) standardized diagnostic criteria for research applications and definitive diagnosis (Fig. 2).

Recommendations for Sarcopenia Assessment: (1) Diagnostic Framework: We endorse the EWGSOP2 or AWGS criteria for early clinical screening and diagnosis of sarcopenia. (2) Community Screening: For population-based preventive screening, grip strength and physical performance measures alone serve as adequate preliminary indicators. (3) Diagnostic Confirmation: Muscle mass quantification remains the cornerstone for definitive sarcopenia diagnosis. Standardized measurement techniques should be employed to ensure data accuracy for both clinical diagnosis and research enrollment.

### The evaluation of muscle strength

The primary parameters for the evaluation of sarcopenia encompass both muscle strength and muscle mass, each of which serves as a critical diagnostic criterion. The European Working Group 2 on Sarcopenia in Older People (EWGSOP2) recommends a structured, stepwise diagnostic framework to effectively determine the presence of sarcopenia in patients.^1^

To assess muscle strength, the measurement of grip strength is often considered a straightforward and convenient methodology. Nevertheless, the reliability of grip strength measurements can be significantly affected by variations in the equipment used and the specific measurement protocols employed. It is strongly recommended that a standardized testing protocol be adopted, incorporating the use of calibrated handheld dynamometers under rigorously defined conditions, supplemented by reference data from a representative population to ensure the precision and accuracy of the results.^23^ This evaluation requires participants to rise from a seated position in a chair and return to a seated posture without using their arms to assist. The time taken to complete five consecutive sit-to-stand cycles should be measured, or alternatively, the number of sit-to-stand repetitions performed within a 30-second interval can be recorded.^1,24^ This test has been demonstrated to yield valid, reliable, and effective indicators of lower limb strength, making it a valuable tool in sarcopenia assessment.^25^

### The evaluation of muscle mass

The evaluation of muscle mass is predominantly performed using imaging techniques, with Dual-energy X-ray absorptiometry (DXA) being the most frequently utilized method. Magnetic resonance imaging (MRI) and computed tomography (CT) are widely regarded as the gold standards for non-invasive assessments of both qualitative and quantitative changes in body composition, including muscle mass and muscle quality.^24,26^ Muscle mass may be expressed as skeletal muscle mass (SMM), appendicular skeletal mass (ASM), or the cross-sectional area of specific muscle groups or anatomical regions. Nonetheless, the widespread application of these methods is constrained by challenges related to accessibility, high costs, non-portability of devices, and the need for highly specialized personnel, thus limiting their feasibility in primary healthcare settings.^24^

We systematically reviewed the assessment criteria and diagnostic cut-off values endorsed by four major consensus groups (EWGSOP2, IWGS, FNIH, and AWGS).^1,20–22^ Additionally, we evaluated the strengths and limitations of these commonly used methods (Table 1). The diagnostic cut-off variations between working groups primarily reflect two factors: the temporal evolution of evidence (with earlier consensus like IWGS/FNIH relying on less comprehensive data) and population heterogeneity due to geographic, ethnic, and sociocultural influences in sampled cohorts.

**Table 1:** Assessment methods for sarcopenia and standardization indicators, advantages and disadvantages

	Examination	Evaluation of indicators	Evaluation criteria	Advantages	Disadvantages
Sift	ARC-F	/	A score of 4 or higher is predictive of sarcopenia and associated adverse outcomes.	For community screening of people at early risk for sarcopenia	Some subjectivity; less precision
	Poll				
Muscular Strength	Grip strength	/	Male <27 kg；female <16 kg (EWGSOP2)	Simple and easy to operate; reliable indicators available	Less accurate; susceptible to other diseases
			Male <28 kg；female<18 kg (AWGS)		
			Male <26 kg；female <16 kg		
			(FNIH)		
	Repeat chair stand test	5 chair stand tests	>15 s (EWGSOP2)		
			≥12 s (AWGS)		
Muscle mass	DEXA	ASMI = ASM/height^2^	Male < 7.0 kg/m^2^	Relatively low cost, low radiation, more accurate and stable differentiation of LM, FM and other indicators	There is a degree of underestimation of sarcopenia compared to CT
			Female < 5.5 kg/m^2^		
			(EWGSOP2)		
			Male <7.0 kg/m^2^;		
			Female <5.4 kg/m^2^ (AWGS)		
			Male <7.23 kg/m^2^; female <5.67 kg/m^2^ (IWCS)		
		ASM adjusted for BMI (ASM_BMI_)	Male <0.789		
			Female <0.512		
	BIA	ASMI	Male <7.0 kg/m^2^ female <5.7 kg/m^2^ (AWGS)	Simple to operate; affordable equipment; widely	There is a degree of error in the conversion equation; the raw measurements do not form a normalized standard
				Available and portable	
	CT	Cross-sectional area CSA of mid-thigh muscles	Male <84 cm^2^	The gold standard for non-invasive assessment of qualitative and quantitative changes in body composition and muscle quantity/mass	Radioactive; high cost of access, lack of portable equipment; need for highly specialized personnel
			Female <84 cm^2^		
		Cross-sectional area of the psoas muscle	SMI male 52–55 cm^2^/m^2^		
			Female 39–41 cm^2^/m^2^		
			SMI male 52.4 cm^2^/m^2^		
			Female 38.5 cm^2^/m^2^		
	MRI	Evaluation site similar to CT	/	Same as CT; non-radioactive; superior for assessment of muscle mass abnormalities such as muscle destruction, abnormal edema, fatty tissue infiltration, etc.	High cost of access; lack of portable equipment; need for highly specialized personnel; no standardized reference data and thresholds yet established
	US	MV, MT, CSA, etc.	/	Low cost, easy to perform, repeatable measurements; non-invasive	Standardized reference data and thresholds not yet established
Physical function	Gait speed	/	Single cut-off speed ≤0.8 m/s (EWGSOP2, FNIH)	Fast test; no special equipment or training required; better suited as a routine medical screening test	Less accurate; susceptible to other diseases
			6 m walking speed <1.0 m/s (AWGS)		
			4-m walking speed <1.0 m/s (IWGS)		
	SPPB	/	≤8 points (EWGSOP2) ≤9 points (AWGS)		
	TUG	/	≥20 s		

*DEXA* Dual-energy X-ray absorptiometry, *CT* computed tomography, *BIA* Bioelectrical impedance analysis, *MRI* Magnetic resonance imaging, *ASM* Appendicular Skeletal Muscle Mass, *SPPB* Simple Physical Performance Battery, *TUG* Timing and advancement test

#### Dual-energy X-ray absorptiometry (DEXA)

Dual-energy X-ray absorptiometry (DXA) is currently recognized as the most widely used technique for assessing whole-body muscle mass. DXA facilitates the evaluation of muscle mass at both the whole-body and regional levels, making it a valuable tool in the clinical assessment of sarcopenia. The technique involves a whole-body scan utilizing an X-ray source emitting at two distinct energy levels (40 and 70 keV).^27^ The radiation dose from a full-body scan is typically around 5 μSv, rendering DXA a particularly safe and viable option for repeated body composition analyses.^27^ Among various imaging modalities, DXA stands out for being relatively cost-effective, offering low radiation exposure, and providing highly accurate and consistent differentiation between lean body mass (LBM), fat mass (FM), and bone mineral content (BMC) at both the regional and systemic levels.^26,28^ In clinical practice, dual-energy X-ray absorptiometry (DXA) typically measures appendicular skeletal muscle mass (ASM), calculated as the sum of lean muscle mass in both upper and lower extremities. To account for interindividual variability, ASM is commonly normalized through three adjustment methods: 1) height-squared adjustment (ASM/height^2^), 2) weight adjustment (ASM/weight), or 3) BMI adjustment (ASM/BMI).^29^ Among these, the height-squared adjusted index (ASMI = ASM/height^2^) has emerged as the standard reference parameter for DXA-based sarcopenia assessment. The revised EWGSOP2 guidelines recommend ASMI thresholds of <6 kg/m^2^ for women and <7.0 kg/m^2^ for men as diagnostic cut-offs for low muscle mass in European populations, as part of the updated criteria for hypomuscular dysfunction.^1^ For the Asian population, the Asian Working Group for Sarcopenia (AWGS) defines the ASMI thresholds similarly, with values of <6 kg/m^2^ for women and <7.0 kg/m^2^ for men.^22^ Numerous studies have demonstrated strong correlations between DXA-derived lower extremity skeletal muscle mass and measurements obtained via CT and MRI. However, it is important to note that DXA tends to slightly underestimate sarcopenia prevalence compared to these more advanced imaging techniques.^30,31^

#### CT

Computed tomography (CT) is extensively utilized for tumor staging and follow-up in various diseases, allowing for sarcopenia assessment in both prospective and retrospective analyses without necessitating additional scans.^27^ CT-based evaluation of muscle mass typically involves measuring the cross-sectional area of muscle from a single CT slice, differentiating between tissues based on X-ray attenuation values, with muscle tissue identified through a standardized range of attenuation (−29 to +150 HU).^32–34^ There are two common methods for muscle mass assessment: one measures the combined cross-sectional area of the paraspinal, psoas major, and abdominal muscles in a single abdominal CT slice at the L3 level, while the other assesses the bilateral psoas major muscles at the L3 and L4 vertebral levels.^34^ CT provides precise visualization of muscle density, and reduced muscle density is commonly associated with increased fat infiltration.^27^ Consequently, CT serves as a valuable diagnostic tool for routine evaluation of both muscle mass and volume.

The cross-sectional area of the psoas muscle at the L3 vertebral level and the mid-thigh muscle area are robust indicators of whole-body skeletal muscle mass. CT scans of specific lumbar vertebrae, particularly L3, show strong correlations with whole-body muscle mass and are frequently used to identify sarcopenia. In their systematic review, Behrang Amini and colleagues concluded that the most frequently measured muscle group in CT assessments is the abdominal wall musculature (142 of 330 participants).^35^ The most commonly used anatomical landmark for muscle mass measurements in CT scans is the L3 vertebra (123 of 142 participants).^35^ The skeletal muscle index (SMI) is the most frequently utilized metric for quantifying abdominal wall muscle mass (114 of 142 participants).^35^ The study reported that the critical range for SMI at the L3 level, based on abdominal CT scans, aligns closely with the findings of Daly et al.^36^ Combining the results, the typical SMI thresholds for sarcopenia were 52–55 cm^2^/m^2^ in men and 39–41 cm^2^/m^2^ in women.^35^ However, given variations in the precise anatomical definitions across different studies, further research is warranted to evaluate the impact of measurement site variations on muscle mass indices (SMI) and indicators of myopathy, such as muscle area (MA), intermuscular adipose tissue (IMAT), and low-density lean tissue. Notably, muscle mass measurements at L3 have been found to differ significantly from those at other vertebral levels. Therefore, the L3-based sarcopenia thresholds are applicable primarily to patients undergoing abdominal CT scans, excluding those receiving only thoracic scans (e.g., for lung cancer screening) or pelvic-only imaging (e.g., for hip fractures).^37^ Additionally, Brian A. Derstine and colleagues evaluated skeletal muscle mass across vertebral levels from T10 to L5.^37^ When L3 is unavailable, they recommend using L2, L4, L5, L1, T12, T11, or T10 in that order of preference.

Mid-thigh CT scans can be used to measure skeletal muscle (SM), intermuscular adipose tissue (IMAT), and subcutaneous adipose tissue (SAT) areas.^38,39^ CT imaging of these regions offers a more comprehensive assessment of muscle mass loss, muscle weakness, and overall muscle function. However, the precise anatomical localization of these measurement sites can vary. For instance, the L3 vertebral level can refer to the upper, middle, or lower portion of the vertebra. Similarly, definitions for mid-thigh CT cross-sections can vary, including measurements taken at the midpoint between the greater trochanter and the intercondylar fossa,^40^ the femur midpoint,^41^ the distal 20 cm of the greater trochanter.^42^ Or the midpoint between the femur and lateral condyle,^43^ etc.^35^ Further research is needed to assess how variations in measurement site localization affect cross-sectional area (CSA), muscle area (MA), intermuscular adipose tissue (IMAT), and other parameters related to muscle mass and fat infiltration.

#### MRI

Magnetic resonance imaging (MRI) operates by detecting the absorption and emission of radiofrequency energy by hydrogen nuclei. The absence of ionizing radiation is a key advantage of MRI, making it particularly well-suited for long-term monitoring of disease progression and treatment outcomes. MRI techniques are primarily employed to evaluate adipose tissue distribution and quantity, and to a lesser extent, skeletal muscle mass.^44^ It is widely regarded as the most advanced imaging modality for characterizing muscle mass loss. MRI allows for a comprehensive evaluation of body composition, revealing abnormalities such as muscle disruption, abnormal edema, fatty infiltration, and fibrous connective tissue formation (myofibrosis).^45,46^ One of the key strengths of MRI is its ability to detect both compositional and structural changes in skeletal muscle associated with aging and disease progression, including decreased muscle contractility accompanied by increased intramuscular adipose (IMAT) and connective tissues (IMCT).^26,45^ The anatomical sites assessed by MRI are similar to those evaluated by CT, with common focus areas being the psoas and mid-thigh muscles.^47^ Studies have indicated that the lumbar L3 vertebral level is optimal for assessing total tissue volume of skeletal muscle (SM), visceral adipose tissue (VAT), and subcutaneous adipose tissue (SAT), whereas mid-thigh muscle measurements show a stronger correlation with total body muscle volume than lumbar muscle measurements from L1 to L5.^48^

The Dixon sequence is highly effective at separating fat from water, providing an accurate quantitative assessment of sarcopenia. Chemical shift-based water/fat separation imaging and dual- or multipoint Dixon sequences are commonly employed techniques for quantifying muscle fat fraction. These methods exploit the differences in resonance frequencies between fat and water protons, enabling precise differentiation and quantification of fat content through careful adjustment of echo time.^32^ In addition to these, various MRI sequences, including T1-weighted imaging, Dixon sequences, T2 mapping (T2 relaxation times), diffusion-weighted imaging (DWI), and non-proton MRI, can be applied for a multifaceted evaluation of muscle mass.^29,49^ However, the current use of these advanced sequences remains predominantly in the realm of research.

Despite the clear advantages, MRI, alongside CT, is regarded as the gold standard for non-invasive body composition and muscle mass evaluation.^24,26^ Nevertheless, the widespread use of MRI in primary care is hindered by high equipment costs, lack of portability, and the requirement for highly specialized operators.^24^ As a result, MRI remains largely confined to research applications. The absence of standardized imaging protocols, normal reference data, and consistent thresholds, along with ambiguities in image segmentation and analysis, continues to limit its clinical utility.^29,49^

#### Ultrasound (US)

Ultrasound (US) is a non-ionizing imaging technique that offers detailed visualization of muscle morphology and size, as well as surrounding structures, allowing for dynamic assessment of soft tissue in real time.^50^ The primary parameters measured by US include muscle volume (MV), muscle thickness (MT), pennation angle (PA), fascicle length (Lf), echo-intensity (EI), and cross-sectional area (CSA).^50,51^ Studies have demonstrated that the use of US to assess muscle thickness and volume yields results that are not significantly different from those obtained via MRI.^34,52,53^ Ultrasound is cost-effective, easy to perform, and highly reproducible, making it an ideal screening tool for sarcopenia, especially in community settings or hospitals. Moreover, three predictive equation models, based on US measurements, have been proposed by two studies and have shown efficacy in assessing muscle mass.^53,54^ The accuracy of these models has been confirmed through comparisons with DEXA measurements, showing no significant statistical difference (*r*^*2*^ = 0.96; *r*^*2*^ = 0.929, standard error of estimate = 2.5 kg; *r*^*2*^ = 0.955, standard error of estimate = 2.0 kg). However, the validity of these ultrasound-based predictive equations requires further verification, given the limited experimental data currently available.

As a relatively recent method for muscle mass assessment, ultrasound has demonstrated considerable promise in providing accurate measurements of muscle mass. It holds potential to become a more precise and accessible diagnostic tool for sarcopenia. Efforts are underway to standardize ultrasound techniques for assessing muscle mass.^51^ Unfortunately, a global consensus on the use of ultrasound for this purpose has yet to be established.

#### Bioelectrical impedance analysis

Bioelectrical impedance analysis (BIA) utilizes equations that calculate differences in electrical conductivity between various tissues to evaluate body composition, including muscle, fat, and bone. The accuracy of BIA is highly dependent on proper tissue hydration.^55,56^ BIA is simple, affordable, widely accessible, and portable, and has been endorsed by both Asian and European guidelines as an effective tool for assessing muscle mass in sarcopenia.^1,57^ Due to these advantages, BIA is increasingly being considered a practical alternative to dual-energy X-ray absorptiometry (DEXA). Multi-frequency BIA, which employs both low and high frequencies to calculate intercellular, extracellular, and total body water, has demonstrated superior accuracy over single-frequency BIA, especially in populations with obesity or underweight conditions.^56^ BIA relies on conversion equations to estimate muscle mass, a process influenced by factors such as age, ethnicity, and the type of measurement equipment used.^58–60^ Recent studies have begun to investigate the development of age-independent conversion equations.^60^ However, the impact of ethnic variability on these equations remains unresolved.Certain equations, like the Sergi equation for adult Australians (Caucasians) and the Kyle equation for male muscle mass assessment, have proven effective in specific populations.^58^ Further research is necessary to validate these prediction equations across diverse populations. It has been observed that BIA tends to overestimate skeletal muscle mass compared to DEXA measurements, particularly in older adults.^61–63^ This discrepancy may be attributed to the fact that the study population was primarily elderly or that the selected BIA methods or equations were suboptimal.^64^

To mitigate errors from BIA prediction equations, researchers have shifted focus to the raw bioelectrical parameters measured by BIA, such as resistance (R), reactance (Xc), and phase angle (PhA)These fundamental bioelectrical measures, particularly PhA, have shown promise in predicting disease prognosis, mortality, and other clinical outcomes.^65,66^ PhA has been found to negatively correlate with muscle mass and strength in older adults, suggesting it could serve as a valuable bioelectrical marker for assessing sarcopenia risk. Nevertheless, further exploration is needed to clarify the relationship between PhA’s predictive capabilities and the diagnosis of sarcopenia.^67–69^

### The evaluation of physical functioning

Physical function, as defined by the European Society for Clinical and Economic Aspects of Osteoporosis and Osteoarthritis (ESCEO),^70^ refers to “an objectively quantified measure of whole-body performance pertaining to mobility”. This concept encompasses multiple dimensions, particularly focusing on both muscular and neural function. In the absence of comorbid conditions that precipitate declines in physical functionality, such as dementia, gait instability, or balance impairments, physical function is typically assessed through various objective measures, including gait speed, the Short Physical Performance Battery (SPPB), physical functioning scales, and the Timed-Up and Go (TUG) test.^1^

Gait speed serves as a highly reliable and sensitive indicator of daily physical activity, and it exhibits a strong correlation with frailty and survival outcomes in elderly populations.^71^ It is widely regarded as a rapid, safe, and highly reliable diagnostic tool for sarcopenia, frequently implemented in clinical practice.^1^ Gait speed is extensively utilized in evaluating physical function within community-dwelling older adult populations and has demonstrated predictive value for adverse outcomes linked to sarcopenia, including falls, frailty, cognitive decline, disability, and mortality.^72,73^ The Short Physical Performance Battery (SPPB) provides an objective and comprehensive assessment of lower extremity function in older adults, incorporating evaluations of gait speed, balance, and the ability to perform chair stands.^74^ The Timed-Up and Go (TUG) test, another widely used tool for assessing physical function in community-dwelling older adults, has been validated as a reliable and effective measure of functional mobility.^75,76^ This test can be seamlessly integrated into routine physical examinations; it is expedient and requires neither specialized equipment nor extensive training.^75^

Screening methods for sarcopenia within community settings must be simple, rapid, and user-friendly. Specifically, the SARC-F questionnaire is recommended in numerous guidelines as a preferred tool for early sarcopenia screening and for identifying individuals at elevated risk.^1,22,77^ Moreover, the 2019 Asian Working Group for Sarcopenia (AWGS) guidelines recommend the use of calf circumference (CC) as a screening metric for sarcopenia, setting reference thresholds at <34 cm for men and <33 cm for women.^22^

### Drugs for sarcopenia

#### Non-pharmacological treatment

Insufficient physical activity results in muscular atrophy, functional deterioration, and increased adiposity. Both lean body mass and fat mass influence bone mineral density, highlighting the critical role of physical activity in preventing bone demineralization. Resistance training, either alone or in combination with aerobic and balance exercises, is considered the most effective intervention for enhancing the quality of life in individuals with sarcopenia.^78^ Studies have demonstrated that resistance exercise stimulates muscle protein synthesis, thereby promoting hypertrophy, increasing muscle strength, and enhancing physical performance.^79,80^ Furthermore, maintaining sufficient physical activity can prevent or mitigate muscle loss by preserving insulin sensitivity and augmenting mitochondrial function.^81^ Endurance exercise promotes mitochondrial biogenesis by upregulating peroxisome proliferator-activated receptor gamma coactivator-1 alpha (PGC-1α), thereby improving skeletal muscle adaptability and overall function.^82–84^ A 5-year longitudinal study involving 1 863 older adults (aged 70–79) in the United States revealed that sustaining moderate physical activity—defined as at least 150 min of moderate-intensity exercise per week, such as brisk walking—significantly lowers the risk of developing or exacerbating metabolic syndrome.^85^

#### Nutritional supplementation

Nutritional supplementation is believed to enhance various physical function outcomes in older adults and those suffering from conditions like frailty or muscle wasting, particularly when incorporating multiple nutrients.^86^ The key nutritional components associated with sarcopenia and frailty pathogenesis include: (1) protein/amino acids, (2) vitamin D, (3) polyphenols (e.g., catechins and isoflavones), (4) antioxidant nutrients (carotenoids, selenium, vitamins E and C), (5) ursolic acid, and (6) long-chain polyunsaturated fatty acids.^87^ Dietary protein provides essential amino acids necessary for muscle protein synthesis. Notably, leucine—a branched-chain amino acid (BCAA)—plays a critical regulatory role by enhancing mitochondrial biogenesis and function in skeletal myocytes.^88^ This mechanism may help mitigate age-related muscle degeneration in both physiological aging and pathological conditions. The Asian consensus guidelines on sarcopenia recommend oral nutritional supplementation (ONS) with high-quality protein, specific amino acids (including leucine and L-carnitine), or β-Hydroxy-β-methylbutyrate (HMB) - a bioactive leucine metabolite.^89^ This leucine metabolite enhances muscle protein synthesis through mTOR activation and anti-apoptotic effects.^90^ While proven effective for maintaining muscle mass in aging adults, HMB’s benefits for strength and physical function require further validation.^91^ Omega-3 polyunsaturated fatty acids (PUFAs) enhance muscle protein synthesis in older adults through mTOR pathway activation and exhibit anti-inflammatory properties.^92^ However, clinical trial evidence has not demonstrated significant efficacy in attenuating age-related muscle loss. Preclinical evidence indicates isoflavones both reduce skeletal muscle adiposity^93^ and prevent denervation-induced atrophy^94^ in male mice. Clinically, their phytoestrogenic properties may offer protection against postmenopausal sarcopenia in older women.^95,96^

In older adults with sarcopenia, high- to moderate-quality evidence suggests that combining nutritional interventions with exercise exerts a more pronounced effect on grip strength and multiple physical function metrics compared to exercise alone.^78^ The concurrent implementation of resistance training combined with nutritional supplementation is anticipated to yield superior therapeutic outcomes.^97^ In a randomized double-blind controlled trial (*n* = 62 male pairs), catechin supplementation alone or resistance training alone demonstrated modest improvements in muscle mass and strength versus placebo.^98^ Notably, the combined intervention (catechin + resistance exercise) showed significantly greater benefits, including: (1) Increased appendicular muscle mass index. (2) Improved performance on the timed up-and-go test. (3) Enhanced levels of muscle growth factors. Compared to monotherapies, combination therapy with HMB and low-magnitude high-frequency vibration (LMHFV) synergistically increased muscle mass while reducing both total adiposity and intramuscular lipid deposition.^99^ The observed effects are principally regulated by activation of the canonical Wnt/β-catenin signaling cascade.

#### Pharmacotherapy

Currently, no pharmacological treatments have been specifically approved for the management of sarcopenia. Medications commonly recommended for potential use include growth hormone (GH), anabolic-androgenic steroids, selective androgen receptor modulators (SARMs), estrogens, protein anabolics, vitamin D, appetite stimulants, myostatin inhibitors, activin II receptor antagonists, beta-blockers, angiotensin-converting enzyme inhibitors (ACE inhibitors), angiotensin receptor blockers (ARBs), and troponin activators, among others.^100,101^

Vitamin D plays a critical role in regulating bone metabolism, particularly in maintaining phosphorus and calcium homeostasis. In the elderly population, approximately 50% exhibit reduced vitamin D levels, primarily due to an age-related decline in vitamin D receptor expression in skeletal muscle. Observational studies have demonstrated a positive correlation between lower vitamin D levels and decreased muscle strength and mass in older adults.^102^ However, a recent systematic review of randomized controlled trials (RCTs) on vitamin D monotherapy for sarcopenia found no significant beneficial effects of supplementation on muscle mass or strength in older adults aged 50 years and above.^103^ Compared with placebo, vitamin D monotherapy demonstrated no significant improvement in grip strength (HGS), gait speed (assessed by timed up-and-go test, TUG), appendicular lean mass (ALM), overall muscle strength, or physical performance in older adults. Notably, vitamin D supplementation was associated with a significant reduction in Short Physical Performance Battery (SPPB) scores.

Myostatin (MSTN), a transforming growth factor-β (TGF-β) superfamily member, negatively regulates skeletal muscle growth and induces atrophy through Smad2/3-dependent signaling pathways.^104^ The humanized monoclonal antibody LY2495655 (LY) specifically targets and neutralizes myostatin, demonstrating efficacy in increasing lean body mass while potentially enhancing muscle strength and physical function.^105^ In clinical trials involving elderly participants, 24-week LY treatment significantly improved key functional outcomes, including stair-climbing capacity, chair-stand performance, and walking speed. Se-Jin Lee comprehensively reviewed current clinical applications of myostatin (MSTN) inhibitors, categorizing them into two classes: (1) relatively specific MSTN inhibitors (including MYO-029, domagrozumab, LY2495655, REGN1033, AMG-745/PINTA-745, BMS-986089/RO7239361, and SRK-015), and (2) broader-spectrum agents targeting MSTN, GDF-11, and activin A (including bimagrumab and ACE-031/083).^106^ Clinical trial evidence demonstrates that these inhibitors (particularly bimagrumab and ACE-031) significantly increase thigh muscle volume and show clinically meaningful effects on overall muscle mass.^107^ Most clinical trials observed that while MSTN inhibitors increased muscle mass, these gains did not translate proportionally to measurable improvements in muscle strength or physical function. This differential response may reflect the high prevalence of comorbidities in elderly populations. Importantly, therapeutic efficacy appears particularly limited in patients with inflammatory myopathies, malignancies, chronic obstructive pulmonary disease (COPD), or end-stage renal disease (ESRD), who showed minimal clinical response to MSTN inhibition. Emerging clinical trial evidence demonstrates that MSTN inhibitors exert dual metabolic benefits, significantly reducing adipose tissue mass while improving glucose homeostasis.^108^ These pleiotropic effects suggest broader therapeutic potential, particularly for managing complex metabolic-muscle disorders such as sarcopenic obesity or multi-morbidity-associated sarcopenia. Future investigations should systematically evaluate these multidimensional therapeutic applications through targeted clinical studies.^109^

Clinical evidence demonstrates that sterol supplementation effectively attenuates age-related declines in muscle mass and grip strength while increasing lean leg mass and limb strength in community-dwelling elderly males.^110^ However, these therapeutic benefits must be weighed against significant adverse effect profiles, including dermatological manifestations (acne, seborrhea), obstructive sleep apnea, thromboembolic events, and potential oncological risks (particularly prostate cancer).^111^

Selective androgen receptor modulators (SARMs), including enobosarm (GSK2881078), demonstrate tissue-specific anabolic activity with favorable pharmacokinetic profiles.^112^ These agents effectively promote muscle hypertrophy while mitigating the adverse effects associated with conventional steroidal therapies.^112^ Preliminary data suggest that oxymetholone increases fat-free mass, grip strength, and physical performance metrics, along with enhancing type I muscle fiber cross-sectional area. However, its clinical application remains limited by hepatotoxicity risks, particularly in hemodialysis-dependent patients.^113^

Current pharmacotherapies for sarcopenia demonstrate limited efficacy in improving physical function and activities of daily living, with variable responses across patient populations. Furthermore, many of these interventions are associated with clinically significant adverse effects. These limitations, coupled with the paucity of large-scale clinical trials, have hindered the widespread clinical adoption of pharmacological interventions for sarcopenia management. Yves Rolland et al.^101^ have systematically reviewed the clinical efficacy of these pharmacological agents, which may have ameliorative effects on muscle loss. Overall, the currently available pharmacotherapies for sarcopenia demonstrate efficacy in enhancing muscle mass and/or strength, yet fail to produce clinically meaningful improvements in physical performance.^101^ Notably, mitochondrial-targeted therapies are gaining increasing research attention. As a representative example, the nicotinic acid derivative acipimox enhances mitochondrial function in human skeletal muscle cells through NAD^+^ biosynthesis upregulation.^114^ The Mitochondria-Targeting Agent MitoQ demonstrates therapeutic potential in murine cancer cachexia models, where it enhances muscle strength and mass, stimulates β-oxidation, and induces a metabolic shift from glycolytic to oxidative fiber types.^115^ However, current evidence remains limited to preclinical studies utilizing animal and cellular models.

## Sarcopenia and osteoporosis

### Clinical relevance

Muscle and bone loss are prevalent among older adults, with the World Health Organization defining osteoporosis as a skeletal disorder marked by compromised bone strength and heightened fracture risk.^116,117^ Recently, the term “Osteosarcopenia” has been introduced to describe the concurrent manifestation of osteopenia or osteoporosis alongside sarcopenia.^118^ Osteoporosis deteriorates bone microarchitecture and diminishes bone strength, whereas sarcopenia is typified by a progressive decline in muscle mass and functionality throughout the body.^119^ These two conditions share several common risk factors—including age, gender, genetic predisposition, metabolic factors, and mechanical stimuli—and are strongly linked to adverse outcomes such as frailty, falls, fractures, hospitalizations, and mortality, significantly increasing the healthcare burden.^120,121^

A multitude of clinical studies have confirmed a robust association between sarcopenia and osteoporosis. A study involving 288 elderly individuals in Belgium revealed a fourfold increase in the risk of comorbid osteoporosis in sarcopenic patients compared to their non-sarcopenic counterparts (OR = 4.18; 95% CI: 1.92–9.12).^122^ An Australian study further suggests that individuals with concurrent sarcopenia and osteoporosis are at a greater risk for falls and fractures than those with either condition alone.^123^ A Korean study of 324 patients with hip fractures found that 93 (28.7%) were diagnosed with osteomuscular decompensation, and this group exhibited a significantly higher 1-year mortality rate (15.1%) compared to those with osteoporosis alone (5.1%) or sarcopenia alone (10.3%).^124^

Chen and colleagues conducted a comprehensive summary of the global epidemiological landscape of sarcopenia, reporting an overall prevalence of 18.5% based on data from 63 369 subjects across 63 studies.^125^ The estimated prevalence of sarcopenia exhibits considerable variation due to heterogeneity in population characteristics, geographic regions, age, sex, study design, diagnostic criteria, and clinical settings. Regionally, sarcopenia prevalence was notably higher in Oceania (22.9%), Africa (21.6%), and South America (20.8%) compared to Europe (10.7%).^125^

### Mechanisms of occurrence

#### Mechanical stimulus

Mechanical stimulation occurs when muscles attach to bones to generate movement, providing the necessary strain to maintain optimal bone health.^120^ Increased muscle mass induces stretching of the periosteum and collagen fibers when mechanical forces exceed a certain threshold, thereby promoting bone growth.^119,126^ During human growth and development, muscles, which precede skeletal growth, help shape the contours of the skeleton and drive periosteal bone expansion, adjusting skeletal density to support the necessary load-bearing capacity.^127–129^ Numerous studies have demonstrated a strong association between the loss of muscle and bone mass and the aging process.^130^ A 4-year longitudinal study of an elderly Japanese population reported a high co-prevalence of osteoporosis (57.8%) among individuals with sarcopenia (*n* = 1 099, prevalence of 8.2%).^131^ The study found that individuals with sarcopenia exhibited reduced bone density, while those with osteoporosis had diminished muscle mass and function.^131^ The study concluded that osteoporosis (OP) may elevate the short-term risk of sarcopenia (SP), suggesting that osteoporosis could be a predictor of future sarcopenia risk.

#### Musculoskeletal crosstalk

The relationship between bone and muscle extends beyond mechanical coupling. Notably, muscle mass in distal extremities correlates with cortical bone thickness, suggesting the existence of systemic paracrine/endocrine signaling between these tissues.^132^ Emerging evidence has identified multiple molecular pathways mediating this biochemical crosstalk within the musculoskeletal unit.

Bones and muscles are interconnected not solely through mechanical stimulation. Muscle mass in distal extremities is also correlated with cortical bone thickness, indicating potential paracrine or endocrine crosstalk that further links muscle and bone. The musculoskeletal system has been shown to communicate through autocrine, paracrine, and endocrine signaling, with multiple pathways identified in this integrated system.^133–135^ Bone can receive anabolic signals from muscle, with several myokines—such as myostatin (muscle growth inhibitor), insulin-like growth factor-1 (IGF-1), interleukin-6 (IL-6), IL-15, irisin, fibroblast growth factor 2 (FGF2), and matrix metalloproteinase 2 (MMP2)—being upregulated during muscle contraction and contributing to both bone formation and resorption.^135–138^ Conversely, prostaglandin E2 (PGE2) and Wnt3a, secreted by osteoblasts, along with osteocalcin (OCN) and IGF-1 from osteoclasts, and sclerostin from both cell types, may regulate skeletal muscle cells.^135^ Hormones critical in the development of osteomalacia include growth hormone/insulin-like growth factor-1 (GH/IGF-1) and sex steroids.^119,129^ These hormones are also integral to the regulation of both bone and muscle development.^129^

The receptor activator of nuclear factor kappa-B ligand (RANKL) plays a pivotal role in physiological bone remodeling and osteoclast formation, as well as in the activation of osteoclasts under pathological conditions.^139^ This process is mediated through its interaction with the nuclear factor kappa-B (RANK) receptor. Osteoprotegerin (OPG), a decoy receptor for RANKL, protects bone from excessive resorption by competitively binding to RANKL, thereby inhibiting the RANKL/RANK signaling pathway.^140^ Given its critical function in regulating bone metabolism, the RANKL/RANK/OPG axis is indispensable for maintaining bone homeostasis, and dysregulation of this system can contribute to bone-related disorders, including osteoporosis. Evidence suggests that the RANK signaling pathway significantly influences skeletal muscle physiology, with effects that parallel those observed in osteoporosis.^141^ Activation of RANK has been shown to exacerbate muscle atrophy, increase fatigue susceptibility, and promote a shift toward fast-twitch fiber predominance. RANK signaling modulates skeletal muscle function by impairing the activity of the sarcoplasmic/endoplasmic reticulum Ca^2+^-ATPase (SERCA), a key regulator of intracellular Ca^2^⁺ homeostasis. Pharmacological inhibition of RANKL enhances limb strength and muscle mass while concurrently improving metabolic function, as evidenced by increased insulin sensitivity and muscle glucose uptake.^142^ Additionally, RANKL blockade downregulates the expression of negative regulators of myogenesis (e.g., myostatin) and pro-inflammatory mediators (e.g., protein tyrosine phosphatase receptor-γ, PTPRγ) in skeletal muscle.

The Wnt/β-catenin signaling pathway serves as a critical regulator of bone development and homeostasis.^143^ Emerging evidence demonstrates that Wnt-mediated signaling plays an essential role in modulating osteoblast metabolism, including the stimulation of aerobic glycolysis, glutamine catabolism, and fatty acid oxidation.^144^ Key cytoplasmic components of the Wnt pathway—such as GSK3β, Dishevelled (Dvl), and adenomatous polyposis coli (APC)—along with their regulatory partners (e.g., microtubule-actin crosslinking factor 1, MACF1), represent promising therapeutic targets for the treatment of diverse bone disorders.^145^ In addition, The Wnt/β-catenin signaling pathway plays a crucial role in skeletal muscle development, growth, and regeneration, while simultaneously suppressing intramuscular adipogenesis.^146,147^ Lin et al. comprehensively reviewed the regulatory potential of Wnt/β-catenin signaling in skeletal muscle-bone interactions.^148^ Current evidence clearly establishes the involvement of Wnt/β-catenin signaling, particularly through the Wnt3a-mediated pathway, in musculoskeletal crosstalk. While Wnt4 and Wnt10b have been implicated in muscle-bone communication, their specific roles in age-related musculoskeletal changes require further investigation.

#### Heredity

Muscle and bone originate from somites and share a common mesenchymal progenitor.^149^ Despite their independent development, muscle and bone form secondary relationships through mutual attachment and exhibit a high degree of reciprocal regulation.^126^ Studies have demonstrated that sarcopenia and osteoporosis are governed by distinct genetic factors, and that muscle strength, muscle mass, bone geometry, and bone mass are all significantly influenced by genetic regulation.^149^ Similarly, extensive experimental studies in animals and human cell models have provided evidence of shared genetic pathways regulating both bone and muscle mass. Genetic polymorphisms in the IGF1 gene^150,155^, myostatin (muscle growth inhibitor)^151^, low-density lipoprotein receptor-related protein 5 (LRP5)^152^, and the vitamin D receptor (VDR)^153,154^ have been linked to both bone and muscle loss.^149,155^

Insulin-like growth factor-1 (IGF-1) plays a fundamental role in promoting the proliferation and differentiation of activated satellite cells, while serving as a critical regulator of growth across multiple tissues, particularly in skeletal muscle and bone. These biological effects are mediated through IGF-1 receptor (IGF-1R) phosphorylation, which subsequently activates two key downstream signaling cascades: the Ras/Raf/MEK/ERK pathway and the PI3K/Akt/mTOR pathway.^156^ While the liver serves as the primary source of circulating IGF-1 in humans, skeletal muscle and bone tissues contribute to local IGF-1 availability through autocrine/paracrine secretion.^157,158^ IGF-1 exerts potent mitogenic and differentiation-promoting effects on both osteoblasts and myocytes, with particularly significant actions on muscle satellite cells.^159^ These mechanisms underlie IGF-1’s direct anabolic effects on skeletal muscle and bone tissue.

As a coreceptor in the Wnt signaling pathway, LRP5 plays a pivotal role in mechanotransduction by converting mechanical loading into osteogenic responses through Wnt pathway activation.^160^ Meanwhile, LRP6 serves as a critical regulator of bone homeostasis, modulating both bone formation and resorption processes. Emerging evidence suggests that LRP5 and LRP6 participate in skeletal muscle myogenesis through Wnt pathway regulation, though they exhibit distinct expression patterns of myogenic and synaptic markers.^161^ Notably, LRP5 has been shown to enhance cardiomyocyte proliferation via activation of the AKT/P21 signaling cascade.^162^ However, the current understanding of LRP5/LRP6-mediated muscle regulation remains limited, and their precise molecular mechanisms require further experimental validation.

Genome-wide association studies (GWAS) have progressively identified multiple genes with the potential to co-regulate both bone and muscle. For example, the Mettl21c gene regulates myogenesis and calcium homeostasis in muscle cells, as well as bone cell viability and resistance to apoptosis, through the NF-κB signaling pathway.^163^ The identified dual roles for the GLYAT gene in both bone development and muscle growth, linked to its regulation of glucose and energy metabolism.^164^ Medina-Gomez employed bivariate GWAS to analyze the polymorphic effects of eight loci on total-body lean mass (TB-LM) and total-body less head bone mineral density (TBLH-BMD) in children, including seven loci previously associated with bone mineral density: WNT4, GALNT3, MEPE, CPED1/WNT16, TNFSF11, RIN3, and PPP6R3/LRP5131.^165^ And a newly discovered locus, 17p11.2, has shown a strong association with total body lean mass (TB-LM), potentially due to the influence of the TOM1L2/SREBF1 gene.^165^ The effects of active SREBP-1 and SREBF1 products on osteoblast and myoblast differentiation are well-established.^166,167^ miRNAs expressed by TOM1L2 are implicated in osteogenic differentiation and skeletal muscle development,^168,169^ although the specific role of this gene in the musculoskeletal system remains to be clarified.

The discovery of these polymorphic genes suggests that shared genetic factors between bone and muscle may contribute to the co-occurrence of osteoporosis and sarcopenia in individuals, highlighting the need for systemic treatments targeting both tissues.

### Treatment of osteomyopenia

#### Non-pharmacological treatment

Reduced or insufficient physical activity can result in muscle wasting, functional decline, and increased adipose tissue accumulation. Furthermore, both lean body mass and adiposity are known to influence bone density. Therefore, maintaining regular physical activity is crucial for preventing both muscle and bone loss. Weight-bearing aerobic exercises (e.g., walking, jogging, tai chi) have been shown to attenuate age-related bone loss through mechanical loading effects. In contrast, resistance training (e.g., weightlifting, swimming, cycling) promotes muscle hypertrophy and increases bone mineral density (BMD), with these effects being primarily localized to the specific musculoskeletal regions engaged during exercise.^170^ Resistance exercise (RE) is considered one of the most effective strategies for mitigating osteomuscular degeneration. Clinical studies have shown that progressive resistance exercise promotes osteoclastogenesis and muscle protein synthesis, leading to improvements in bone microarchitecture, muscle mass, strength, and functional capacity in older adults with osteoporosis and sarcopenia.^121^ Mechanistic studies reveal that resistance training specifically elevates serum levels of procollagen type 1 N-terminal peptide (P1NP), a biomarker of bone formation, while simultaneously increasing osteoblast proliferation without corresponding elevation in bone resorption markers.^171^ This dual mechanism demonstrates that aerobic exercise exerts comprehensive skeletal benefits by both suppressing bone catabolism and promoting anabolic activity. Whole-body vibration (WBV) is a therapeutic modality that delivers high-frequency mechanical stimulation via a vibrating platform, targeting bone mechanoreceptors to promote osteogenesis. Clinical evidence indicates that whole-body vibration (WBV) therapy demonstrates significant benefits in improving key physical performance measures in elderly populations, including muscular strength, postural balance, mobility, and gait function.^172^ However, the therapeutic efficacy of WBV for enhancing muscle mass and bone mineral density (BMD) remains inconclusive, with existing studies reporting conflicting outcomes.^173^

Nutritional supplementation has proven equally effective in managing osteomyopenia. Multinutrient supplementation has been shown to improve various physical functioning outcomes in older adults, as well as in individuals affected by specific medical conditions or frailty-related muscle loss.^86^ As previously discussed, low serum vitamin D levels show a significant positive correlation with decreased muscle strength and mass in older adults. While current evidence fails to demonstrate substantial benefits of vitamin D supplementation for sarcopenia management, its critical role in bone health maintenance is well-established. Research indicates that vitamin D supplementation improves bone mineral density (BMD) exclusively in individuals with 25-hydroxyvitamin D [25(OH)D] levels below 30 nmol/L.^174^ Notably, high-dose supplementation (>4 000 IU/day) may paradoxically increase fall and fracture risks.^175^ Consequently, current guidelines do not recommend routine calcium or vitamin D supplementation for osteoporosis prevention in healthy community-dwelling adults without documented deficiencies.^176^ The European Society for Clinical and Economic Aspects of Osteoporosis, Osteoarthritis, and Musculoskeletal Diseases (ESCEO)^177^ advises a daily intake of 800 IU of vitamin D for postmenopausal women, aiming to maintain 25(OH)D levels above 50 nmol/L.

#### Pharmacotherapy

Growth hormone (GH) is a critical molecule that facilitates both bone and muscle development throughout human growth. Its physiological effects are primarily mediated via the GH/IGF-I axis.^178^ The GH/IGF-I axis governs muscle cell proliferation, modulates myofibril size and fiber type, promotes osteoblast proliferation and differentiation, inhibits osteoclast activity, and regulates renal 1α-hydroxylase activity (which activates 25-OH-vitamin D) and phosphate reabsorption.^129^ Despite its potential, recombinant human growth hormone (rhGH) therapy for age-related muscle, bone loss, and adiposity alterations remains a subject of ongoing debate. In a pivotal study involving 12 elderly males treated with rhGH for 6 months, Rudman et al.^179^ observed an 8.8% increase in lean body mass, a 1.6% improvement in lumbar spine bone density, and a 14.4% reduction in adiposity, while femoral neck bone density remained largely unchanged. A 7-year longitudinal study in osteoporotic patients revealed that rhGH replacement therapy demonstrated greater efficacy in the initial 4 years, marked by a substantial increase in lumbar spine BMD, followed by a diminished effect over the subsequent 3 years.^180^

Selective androgen receptor modulators (SARMs) were developed to elicit anabolic effects in muscle and bone, while circumventing the dose-limiting androgenic effects commonly associated with testosterone, such as prostate growth, acne, and oily skin.^129^ Andarine has been characterized as an ideal SARM due to its once-daily administration, complete oral bioavailability, and substantial preclinical evidence documenting its anabolic effects on muscle and bone.^181^ Intervention with the selective androgen receptor modulator (SARM) ostarine in ovariectomized female rats resulted in increased gastrocnemius muscle weight, improved bone biomechanical properties, and enhanced bone mineral density.^182^ Clinical trials have reported improvements in skeletal muscle mass with SARM treatment; however, its effects on muscle strength and function, as well as its long-term efficacy, require further investigation.^183,184^ The U.S. Food and Drug Administration (FDA) has not approved SARM for the treatment of sarcopenia. The U.S. Food and Drug Administration (FDA) has not approved SARMs for the treatment of sarcopenia. However, clinical data on their efficacy and safety continue to emerge, suggesting potential therapeutic roles as anabolic and functional agents in various musculoskeletal disorders.^185^

Emerging evidence suggests that select anabolic agents, including myostatin inhibitors, can simultaneously enhance muscle and bone mass.^186^ Notably, the soluble activin type IIB receptor (ActRIIB-Fc) demonstrated significant efficacy in a murine muscular atrophy model, increasing both limb/axial bone mass and improving long bone biomechanics.^187^ Mechanistic analysis revealed that the osteogenic effects of ActRIIB-Fc were activity-independent, with no correlation observed between bone mass changes and potential alterations in murine locomotor behavior.^188^ These findings position myostatin modulation as a promising therapeutic strategy for musculoskeletal disorders, particularly in patients with limited mobility. However, further preclinical and clinical research is required to establish the long-term safety and therapeutic potential of these compounds.

## Sarcopenia and osteoarthritis

Osteoarthritis is the most common chronic joint disease, which can affect any joint. However, it most frequently targets the knees, hips, hands, facet joints, and feet, involving both cartilage and the surrounding tissues.^189^ Osteoarthritis is primarily characterized by the degeneration and loss of articular cartilage, along with intra-articular bone remodeling, osteophyte formation, ligament laxity, muscle weakness around the joints, and synovitis, etc.^190^ It is mainly manifested as joint pain, stiffness, and limited movement. Several specific risk factors for osteoarthritis have been identified, including obesity, metabolic diseases, age, gender, ethnicity, genetics, nutrition, joint overload or abnormalities, previous injuries, bone density, and muscle function.^190–192^

### The correlation between sarcopenia and osteoarthritis

Mechanical load is undoubtedly a key risk factor in the development of osteoarthritis and is regarded as the only critical factor in its progression.^193^ The risk factors for osteoarthritis are heightened by increased mechanical stress on the joints (such as obesity, excessive joint load, or joint misalignment) or by reducing the joints’ mechanical protection (such as through injury or muscle loss). This weakens the joints’ ability to withstand normal loads, accelerating the onset of osteoarthritis.^193^

Growing evidence suggests that reduced lower limb muscle strength is commonly observed in patients with knee or hip osteoarthritis at various stages. Lower limb muscle weakness is a known predictor for the onset of knee osteoarthritis, though there is conflicting evidence regarding its role in the progression of the disease.^194^ The prevalence of sarcopenia (45.2%) among 7 495 knee osteoarthritis patients (average age 68.5 years, with 72.4% being female) in Asia and Europe is more than twice that of the control group (31.2%), with an odds ratio (OR) of 2.07.^195^ However, obesity continues to play a significant role. In a large sample screening of 11 456 adults in the United States, sarcopenia—defined by BMI-adjusted SMI—was found to be associated with osteoarthritis, with the correlation being stronger in the smoking population.^196^

However, it is worth noting that some studies have not demonstrated a clear correlation between sarcopenia or thigh muscle weakness and osteoarthritis. For example, a study analyzing a population of 1 653 participants with or at risk for knee osteoarthritis found that muscle wasting alone was not associated with an increased risk of knee osteoarthritis, whereas a combination of obesity and muscle wasting was linked to a higher risk.^197^ Alternatively, it only demonstrates a correlation between knee osteoarthritis and high fat mass combined with low lower limb muscle mass in female patients, with no such evidence found in male OA patients.^198^ The discrepancies in the analysis of these clinical data may arise from varying definitions of sarcopenia and the influence of other confounding factors that can contribute to both sarcopenia and osteoarthritis. This highlights the need for more precise definitions of patient subgroups and careful selection of endpoints to better clarify the link between the two conditions.

The relationship between osteoarthritis and muscle loss can be studied longitudinally using animal models in which muscle weakness or osteoarthritis is induced.By using rat anterior cruciate ligament transection (ACLT) to simulate human knee osteoarthritis (KOA)-like changes, it was found that the increased expression of MuRF-1 and atrogin-1, the key signaling molecules of muscle atrophy, may be linked to alterations in the neuromuscular junction (NMJ).^199^ ACLT-induced KOA promotes neuromuscular junction (NMJ) remodeling and atrophy in the quadriceps and tibialis anterior (TA) muscles, which is associated with signs of inflammation and alterations in muscle gene and protein expression.^199^

Due to the current lack of specific biomarkers that can fully validate cartilage degeneration and muscle weakness, along with the absence of unified and effective diagnostic indicators for muscle atrophy, verifying the relationship and underlying mechanisms between muscle atrophy and osteoarthritis has become increasingly complex. Clinical studies have identified muscle weakness as a potential key risk factor for knee osteoarthritis. Patients with osteoarthritis, particularly those with pain symptoms, show lower quadriceps muscle strength compared to the general population, as indicated by imaging evidence.^200^ Unlike typical muscle atrophy, which is often characterized by type II fiber atrophy, the muscle atrophy in osteoarthritis patients shows more uniform fiber involvement. This is primarily associated with the functional impairments caused by the disease.^201^ Further research is needed to explore potential factors such as muscle growth inhibitors, systemic or local inflammatory mediators, cytokines, and inflammatory transcription factors.

### The impact of sarcopenia on the therapeutic effect of osteoarthritis

Sarcopenia and osteoarthritis share many similarities in both physical and pathological manifestations, with correlations ranging from traditional mechanical interactions to endocrine and biochemical signaling.Therefore, exploring common treatment approaches for both conditions, or adjusting the treatment of one to improve the other, is currently a key focus in clinical research.

Currently, the primary drug treatments for osteoarthritis include nonsteroidal anti-inflammatory drugs (such as celecoxib), central analgesics (such as duloxetine), opioid agonists (like tramadol), and intra-articular injections of hyaluronic acid.^192^ The treatment methods mainly aimed at relieving pain in this part are not highly correlated with the treatment of sarcopenia. Non-pharmacological treatments for osteoarthritis, such as weight loss (for overweight individuals) and strengthening exercises, are similar to the interventions used for sarcopenia. Muscle mass and strength serve as crucial links between sarcopenia and osteoarthritis in clinical treatment. Structured exercise interventions targeting lower limb muscle strengthening can help alleviate pain and improve functional status.Clinical studies have shown that combining diet with exercise can significantly reduce weight, alleviate pain, improve functional status, and lower inflammatory markers more effectively than exercise alone.^202^ Liao et al.^203^ found that resistance training combined with protein supplementation can effectively improve muscle mass, strength, and function, enhance joint stability and body balance in osteoarthritis patients at risk of muscle atrophy, thereby reducing pain and improving mobility.

### The impact of sarcopenia on osteoarthritis surgery and prognosis

OA patients with persistent pain, functional loss, and late stage imaging changes may choose to undergo total hip arthroplasty (THA) or total knee arthroplasty (TKA). TKA and THA surgeries positively impact muscle strength and performance in osteoarthritis patients, promoting joint mobility and aiding in recovery. Sarcopenia has been found to affect postoperative rehabilitation outcomes following total joint arthroplasty (TJA). A follow-up study of 90 438 osteoarthritis patients revealed that those with muscle atrophy experienced longer hospital stays, higher risks of medical complications and reoperation within 90 days, and an increased rate of prosthesis failure within 2 years.^204^ A follow-up study of 90 438 osteoarthritis patients found that those with muscle atrophy had longer hospital stays, increased risks of medical complications and reoperation within 90 days, and a higher rate of prosthesis failure within 2 years.^205^ Overall, patients with advanced osteoarthritis, whether or not they have sarcopenia, have shown significant clinical improvements in muscle mass, strength, function, pain, and daily living abilities following TKA surgery.^206^ In addition, TKA surgery improves the muscle strength of the quadriceps and hamstrings in patients with knee arthritis, with gradual recovery approaching the strength of the healthy side at 1, 3, and 6 months post-surgery.^163^

In addition, sarcopenia may increase the risk of prosthetic infection after joint replacement surgery. Babu et al.^165^ used the psoas lumbar vertebral index (PLVI), a marker of central muscle atrophy, to analyze patients with and without prosthetic infections following THA and TKA. They found a significant difference in PLVI between the two groups, with infected patients having significantly lower PLVI. Multivariate logistic regression analysis further indicated that PLVI is an important predictor of postoperative prosthetic infection.

## Sarcopenia and spinal degenerative diseases

### Sarcopenia and scoliosis

Adult degenerative scoliosis (ADS) is a common spinal condition characterized by abnormal curvature of the spine, often resulting in pain, stiffness, and loss of function. The pathogenesis of ADS is primarily attributed to asymmetric degeneration of intervertebral discs and facet joints at various levels.^166^ This asymmetrical pathological change creates an imbalance in spinal load, ultimately leading to the formation of abnormal spinal curvature, which progressively worsens and compromises the spine’s functional integrity. Clinical studies have shown that ~50% of patients with degenerative lumbar scoliosis (DLS) also experience muscle atrophy.^15,167^ Yawara et al.^168^ included 971 women (average age 70.4 years) in their study and found a comorbidity rate of 59.8% between degenerative lumbar scoliosis (DLS) and sarcopenia. Logistic regression analysis identified a reduction in trunk muscle mass as an age-related risk factor for DLS.^168^ Unfortunately, there is currently limited clinical data and research on the comorbidity of muscle wasting in patients with scoliosis. More studies with larger sample sizes are needed to better evaluate the correlation between degenerative scoliosis and muscle wasting.

The onset of degenerative scoliosis is linked to factors such as asymmetric degeneration of intervertebral discs and facet joints, reduction of the muscles surrounding the spine, uneven distribution of mechanical loads, and the degradation of inflammatory factors and the extracellular matrix (ECM).^166^ Sarcopenia may influence scoliosis by disrupting the balance between the spine’s extensor and flexor muscles. Clinical studies have found that the difference in the paraspinal and lumbar muscle area in patients with degenerative scoliosis is significantly greater on the convex side compared to the concave side. This may be due to fat infiltration on both sides and muscle hypertrophy on the convex side.^169^ Another study found that in patients with degenerative lumbar scoliosis (DLS), the multifidus muscle on the concave side exhibited a reduction in muscle fiber size and a decrease in the number of cell nuclei, suggesting that degenerative scoliosis is associated with muscle degeneration on the concave side.^74^

The impact of sarcopenia on scoliosis may also be linked to certain secreted factors. The pathogenesis of sarcopenia involves oxidative stress and chronic inflammation. Oxidative stress, driven by the accumulation of reactive oxygen species, along with inflammatory factors such as IL-6 and TNF-α, can promote mitochondrial dysfunction and further induce muscle cell apoptosis.^182^ High levels of mitochondrial DNA (mtDNA) and mtDNA deletions have been found in the paraspinal muscles of patients with scoliosis, indicating a potential link to muscle atrophy.^183^ Pentosidine, a potential biomarker of sarcopenia and an advanced glycation end product (AGE), has been found in higher serum concentrations in elderly women with degenerative lumbar scoliosis (DLS). It is also associated with the severity of coronary artery disease and sagittal displacement in these patients.^167,184^ This suggests that elevated levels of AGEs could serve as potential biomarkers for the progression of lumbar scoliosis and kyphotic deformities.

### Sarcopenia and intervertebral disc degeneration

Intervertebral disc degeneration (IVDD) is one of the most common clinical health issues, and the progressive decline in paraspinal muscle mass, strength, and function due to sarcopenia (SP) has been identified as a significant factor contributing to IVDD.^185,207^ Qi et al.^208^ explored the causal relationship between sarcopenia-related traits—such as appendicular lean mass (ALM), grip strength (GS), and walking speed (WP)—and intervertebral disc degeneration (IVDD) using a two-sample Mendelian randomization approach. They concluded that severe sarcopenia is a significant risk factor for intervertebral disc degeneration (IVDD). Clinical studies have shown that among 120 IVDD patients, 28.3% also had concurrent muscular dystrophy.^209^

A cross-sectional study has shown a correlation between lumbar disc herniation and paraspinal muscle degeneration, but found no association with muscle asymmetry.^210^ The paraspinal muscles, including the multifidus and erector spinae, are situated on both sides of the spine and play a vital role in maintaining spinal stability and function. These muscles have been shown to be closely associated with the development of various spinal conditions, including lumbar disc herniation, lumbar spinal stenosis, and paraspinal kyphosis.^211–215^ Multifidus muscle biopsies from individuals with lumbar spine lesions have revealed increased levels of muscle degeneration, inflammation, and reduced vascularization.^216^ A retrospective study involving 132 patients with intervertebral disc degeneration (IVDD) and healthy controls identified a potential bidirectional relationship between multifidus muscle degeneration and the progression of IVDD, suggesting mutual influence and interaction between these conditions.^217^ Guangming Xu et al.^218^ conducted a quantitative analysis of the effects of erector spinae and multifidus muscle atrophy on spinal tissue using a human-based finite element (FE) spinal model. Their findings indicated that muscle atrophy primarily increases the risk of damage to the L4-L5 intervertebral discs, L1 vertebrae, and L3-S1 joint capsules, as evidenced by significant stress and strain differences in these areas. Additionally, the intervertebral vacuum phenomenon (IVP) is recognized as one of the imaging markers associated with intervertebral disc degeneration. Camino Willhuber, Gaston et al.^219^ found that patients who underwent lumbar decompression surgery exhibited a higher degree of intervertebral vacuum phenomenon (IVP). The severity of IVP was positively correlated with fat infiltration in the multifidus and erector spinae muscles, with a stronger correlation observed in the multifidus. Adipose degeneration of paraspinal muscles is believed to influence the recovery of patients with lumbar disc degeneration (LDD) undergoing open microdiscectomy, particularly at 1 and 6 months post-surgery. This degeneration may negatively affect the overall recovery process and the success of the surgical intervention.^220^ Additionally, animal studies have been employed to investigate the relationship between intervertebral disc degeneration and paraspinal muscle atrophy. Hey HWD et al.^221^ successfully simulated human paraspinal muscle atrophy using TSC1 gene knockout mice, and their findings revealed that paraspinal muscle atrophy significantly accelerated intervertebral disc degeneration and height loss in the mice.A study using a ram model of intervertebral disc degeneration (IVDD) suggests that back muscle injury resulting from IVDD is characterized by structural remodeling of muscles, fat, and connective tissue, rather than being limited to muscle atrophy alone.^222^

Segmental mechanical dysfunction resulting from defects in the structure and activation of the multifidus muscle may lead to cumulative damage to the annular fibers of the intervertebral discs, a process that has been shown to contribute to intervertebral disc degeneration.^223^ Sven Hoppe et al.^224^ conducted a 3D analysis of paraspinal lumbar muscle fat infiltration using MRI T2-weighted images and identified a positive correlation between the degree of fat infiltration in the paraspinal muscles and the Pfirrmann grade of intervertebral disc degeneration.Clinical studies have shown that the multifidus muscles of patients with disc herniation contain a significant proportion of fibroblast adipogenic progenitor cells (FAPs) and satellite cells (SCs). These cells are believed to be the primary contributors to muscle fibrosis and fat infiltration in these patients.^225^

At the molecular level, signal transduction pathways involving fibroblast growth factor (FGF) have been found to be associated with various musculoskeletal disorders, including intervertebral disc degeneration (IVDD), sarcopenia, osteoarthritis (OA), and osteoporosis (OP).^226^ Numerous fibroblast growth factor (FGF) family members have been shown to regulate the synthesis, catabolism, and ossification of cartilage tissue, while also influencing the regeneration and differentiation of muscle stem cells, myoblasts, and muscle tissue. A study utilizing 3T magnetic resonance imaging found a significant positive correlation between the proton density fat fraction (PDFF muscle) of paraspinal muscle fat content and an increased risk of intervertebral disc degeneration (IVDD) in individuals with impaired blood glucose levels.^207^ This suggests that the relationship between paraspinal muscle atrophy, fat infiltration, and IVDD may be driven, at least in part, by dysregulated blood glucose metabolism in these patients. Increasing evidence suggests that adipose tissue acts as a significant source of pro-inflammatory cytokines. Animal studies have shown that the multifidus adipose tissue in sheep with intervertebral disc degeneration (IVDD) contains a higher proportion of M1 macrophages and elevated expression of pro-inflammatory cytokines, such as TNF. In line with these findings, researchers observed fat infiltration in the multifidus muscle of patients who had undergone surgery for intervertebral disc herniation. They discovered that TNF expression and markers of pro-inflammatory M1 macrophages were elevated in multifidus muscles with high-fat infiltration, confirming that intervertebral disc degeneration is linked to the dysregulation of local inflammatory responses, particularly multifidus myositis.^227^ Additionally, data from 21 patients with lumbar disc herniation who underwent microdiscectomy in Australia showed that impaired regenerative capacity of the multifidus muscle was linked to adverse outcomes post-surgery.^228^ This study also suggested that inflammatory imbalances in subcutaneous fat in the back area may be a contributing factor to these poor outcomes.

Unfortunately, current research on the correlation between IVDD and sarcopenia is mainly based on clinical sample observations, and further exploration of the specific mechanisms is urgently needed.

### Sarcopenia and lumbar spondylolisthesis

Degenerative lumbar spondylolisthesis (DLS) refers to the forward or backward displacement of a vertebral body relative to the adjacent vertebra, caused by degenerative changes, without the presence of a corresponding vertebral ring fracture or defect.^227^ DLS results from the combined influence of multiple factors, including pelvic tilt and misalignment, the strength of the iliac neck ligaments, and degeneration of adjacent intervertebral discs or facet joints. These factors contribute to the instability and displacement of the vertebral body.^229^ Among them, the reduction of paraspinal muscles and fat infiltration are also one of the main reasons.^230,231^ The paraspinal muscles play a critical role in providing functional support and maintaining spinal stability. For example, the multifidus muscle is essential for spinal rotation and stability, while the erector spinae muscle assists with spinal flexion and extension.^231–233^ At the molecular level, factors such as fat infiltration into muscle tissue, insulin resistance, and mitochondrial dysfunction can contribute to muscle atrophy and reduced muscle strength. This decline in muscle function compromises spinal stability, which can ultimately lead to the development of degenerative lumbar spondylolisthesis (DLS).^234^

Cao et al.^233^ observed the fat infiltration rate (FIR) of paraspinal muscles (PM) in patients with pure L4 vertebral body degenerative lumbar spondylolisthesis (DLS) and found that FIR of the paraspinal muscles is an independent factor associated with asymptomatic adult L4 DLS. This finding suggests that FIR has a high predictive value for identifying L4 DLS in these patients. Duan et al.^230^ retrospectively analyzed preoperative MRI scans of 23 patients with spinal spondylolisthesis who underwent single-level L4-5 transforaminal lumbar interbody fusion (TLIF) surgery. They found that the degree of fat infiltration in the multifidus muscle, particularly at the L3 level, was significantly associated with adjacent segment degeneration (ASD) following surgery for degenerative spondylolisthesis. Paul Köhli et al.^235^ examined the functional cross-sectional area (FCSA) and degree of fat infiltration in the psoas major, erector spinae (ES), and multifidus (MF) muscles in patients undergoing surgery for L4/5 level degenerative lumbar spondylolisthesis (DLS). They found a significant correlation between the condition of the paraspinal muscles and the severity of DLS slippage. Specifically, greater degeneration in the ES and MF muscles was associated with a higher degree of slippage, whereas the psoas muscle exhibited an inverse relationship. Another study on the cross-sectional area (CSA) and fat degeneration of lumbar paraspinal muscles in adult DLS patients with chronic nerve root disease found that these patients had smaller MF FCSA and higher fat infiltration in the MF, indicating segmental atrophy of the MF muscles.^231^ This atrophy may be compensated for by ES muscle hypertrophy.^236^ James C McKenzie et al.^237^ compared outcomes between sarcopenia and non-sarcopenia patients who underwent lumbar fusion surgery and found no significant differences in clinical results, suggesting that sarcopenia does not negatively impact the success of lumbar fusion surgery in treating degenerative spondylolisthesis. In another clinical study, preoperative and postoperative MRI data from 95 patients with lumbar spinal stenosis who underwent microsurgical decompression (MSD) were analyzed. The study found that a decrease in lumbar muscle volume, as indicated by the psoas muscle index (PMI), was significantly correlated with the progression of postoperative spondylolisthesis. However, there was no statistically significant correlation between multifidus muscle volume and the progression of postoperative slippage.^238^

### Sarcopenia and thoracolumbar disease

Emerging evidence demonstrates an association between paravertebral muscle degeneration and various spinal pathologies. However, current research has predominantly focused on lumbar spine disorders, with limited investigation into paraspinal muscle changes in cervical and thoracic spine conditions. A critical unanswered question is whether cervical paravertebral muscle evaluation could serve as a reliable surrogate marker for sarcopenia assessment, which would potentially eliminate the need for additional lumbar CT scans and reduce both healthcare costs and patient radiation exposure. In this context, Furkan Ufuk et al. conducted a pioneering study examining the correlation between cervical paraspinal muscle measurements from CT scans and the established L3 muscle index (L3MI), providing preliminary reference values for clinical assessment.^239^ The size of the deep extensor muscles, muscle asymmetry, and the ratio of functional cross-sectional area to total cross-sectional area have been found to influence surgical outcomes in patients with degenerative cervical spondylotic myelopathy (DCM).^240^ Patients with DCM who display increased paraspinal fat infiltration and asymmetry in the deep cervical muscles tend to experience more severe spinal canal damage, as indicated by maximum canal compromise (MCC). Notably, conflicting evidence exists regarding the clinical relevance of cervical paravertebral muscle degeneration. Some studies have failed to demonstrate significant correlations between cervical paraspinal muscle atrophy and fatty infiltration in patients undergoing anterior cervical discectomy and fusion (ACDF).^241,242^

Existing investigations into thoracic spine pathologies and sarcopenia have primarily examined their association with vertebral fractures and osteoporosis. Emerging evidence indicates that the skeletal muscle index (SMI) derived from T12-level CT imaging serves as both a reliable diagnostic marker for osteoporosis and a predictive tool for fracture risk.^243^ Epidemiological data from community-dwelling older adults reveal that sarcopenia constitutes a significant risk factor for moderate-to-severe thoracic fragility fractures, with fracture severity correlating with the degree of sarcopenia.^244^ Biomechanical modeling using the AnyBody simulation system demonstrates maximal spinal loading in older adults occurs at the thoracolumbar junction (T9/T10–L1/L2).^245^ The loss of paravertebral muscle mass increases compressive and shear stresses on osteoporotic vertebrae, likely explaining the elevated fracture risk observed in sarcopenic populations.^246^

## Discussion

In this review, we discuss current assessment methods for sarcopenia and its correlation with common musculoskeletal disorders, particularly age-related degenerative diseases. Currently, standardized diagnostic criteria for sarcopenia remain lacking in clinical practice. Based on existing evidence, we recommend adopting the comprehensive evaluation frameworks proposed by EWGSOP2 and AWGS for sarcopenia diagnosis. The assessment approach should be tailored to specific clinical contexts: For screening purposes, simplified evaluations focusing on muscle strength and physical performance may be employed. However, in research settings or when comorbid conditions may confound functional assessments, imaging modalities remain the gold standard for precise muscle mass quantification. Selection of anatomical regions for evaluation should be guided by clinical objectives. For instance, when investigating lumbar spine disorders with concurrent sarcopenia, the lumbar dorsal muscle group at the L3 vertebral level represents the preferred measurement site, as it provides standardized and reproducible assessment. However, despite the relative accuracy and ease of use of clinical methods like ultrasound and bioelectrical impedance analysis, these techniques still have limitations and lack a standardized evaluation protocol.

Muscle atrophy, a key feature of sarcopenia, is most prevalent in the elderly population and is closely associated with various adverse health outcomes in this group. It has been found to be highly correlated with conditions such as osteoporosis, osteoarthritis, and various spinal disorders.Further exploration into the mechanisms linking sarcopenia and musculoskeletal diseases, as well as the search for common pathogenic factors, may uncover new therapeutic targets for both conditions. Future research should also focus on developing interventions that address both sarcopenia and musculoskeletal disorders. In clinical practice, alongside the treatment of common skeletal and muscular conditions, greater emphasis should be placed on assessing patients’ muscle loss to improve treatment efficacy, restore physical function, and enhance quality of life.

Our work still has certain limitations. Firstly, we selected only a subset of common musculoskeletal disorders, particularly those with a strong correlation to muscle atrophy, and did not include diseases that have received less research attention. Secondly, some of the studies we reviewed may have differing definitions and assessment criteria for muscle atrophy, and sample sizes in these studies could be insufficient. Therefore, the correlation between muscle atrophy and skeletal muscle diseases requires further investigation with larger sample sizes and standardized evaluation methods.
